# A New pH-Responsive Nano Micelle for Enhancing the Effect of a Hydrophobic Bactericidal Agent on Mature *Streptococcus mutans* Biofilm

**DOI:** 10.3389/fmicb.2021.761583

**Published:** 2021-10-18

**Authors:** Meng Zhang, Zhiyi Yu, Edward Chin Man Lo

**Affiliations:** ^1^Faculty of Dentistry, The University of Hong Kong, Pok Fu Lam, Hong Kong, SAR China; ^2^Department of Medicinal Chemistry, School of Pharmaceutical Sciences, Cheeloo College of Medicine, Shandong University, Jinan, China

**Keywords:** pH-responsive micelle, drug delivery systems, hydrophobic antibacterial agent, dental caries, *Streptococcus mutans*

## Abstract

The bactericidal effect on biofilm is the main challenge currently faced by antibacterial agents. Nanoscale drug-delivery materials can enhance biofilm penetrability and drug bioavailability, and have significant applications in the biomedical field. Dental caries is a typical biofilm-related disease, and the acidification of biofilm pH is closely related to the development of dental caries. In this study, a pH-responsive core-shell nano micelle (mPEG-b-PDPA) capable of loading hydrophobic antibacterial agents was synthesized and characterized, including its ability to deliver antibacterial agents within an acidic biofilm. The molecular structure of this diblock copolymer was determined by hydrogen-1 nuclear magnetic resonance (^1^H-NMR) and gel permeation chromatography (GPC). The characters of the micelles were studied by dynamic light scattering (DLS), TEM, pH titration, and drug release detection. It was found that the hydrophilic micelles could deliver bedaquiline, a hydrophobic antibacterial agent on *S. mutans*, in acidic environments and in mature biofilm. No cytotoxic effect on the periodontal cells was detected within 48 h. This pH-responsive micelle, being able to load hydrophobic antibacterial agent, has good clinical application potential in preventing dental caries.

## Introduction

The oral cavity harbors an extremely sophisticated microbial environment in nature. As many as 700 strains live on hard and soft tissue surfaces. Maturation of biofilm and the imbalance of microecology contribute to the development of oral diseases (Gao et al., 2018). Dental plaque biofilm is closely related to the occurrence and development of dental caries (Pitts et al., 2017). Different bacteria colonize the tooth surface in a well-programmed way and form a biofilm (Kolenbrander et al., 2006). This microecology not only provides a great haven for the reproduction of caries pathogens and the fermentation of an acidic environment (Filoche et al., 2010) but also resists attack from foreign substances, including antibiotics and antibacterial agents (Pizzolato-Cezar et al., 2019). Many agents have an antibacterial effect on cariogenic pathogens in suspension (Jiang et al., 2020; Zhang et al., 2021), but it is extremely difficult for these agents to penetrate into biofilm and attack the pathogens in the biofilm. This is a major challenge for the clinical applications of these agents for dental caries management.

Nanoscale drug carriers have been shown to be able to release its cargo in a controlled manner, increase the penetration to biofilms, improve drug stability, and enhance drug bioavailability (Mura et al., 2013; Liu et al., 2016; Mishra et al., 2019). Nanoparticles, as a biofilm-targeting approach, have become increasingly popular due to their versatility and biological activity (Koo et al., 2017). Multidrug-resistant bacterial infection is also expected to be overcome by effective antibacterial agents with the assistance of nanoscale drug carriers (Qing et al., 2019). Based on the properties and advantages of nanoscale materials, they have good potential to be selected as an adjuvant to tackle the abovementioned challenges in dental caries management by optimizing the clinical application of antibacterial agents (Hannig and Hannig, 2010). Some nanomaterial studies have achieved good results on dental surface coating and biofilm penetration. Liu et al. (2016) designed and confirmed a mixed-shell-polymeric-micelle [PEG-poly(β-amino ester)] which enhanced the penetration capability and bactericidal effectiveness of triclosan to *Staphylococcal* biofilms. Another group of researchers (Horev et al., 2015) found that nanomicelles [p(DMAEMA-co-BMA-co-PAA)] loaded with antibacterial agent displayed multi-targeted binding on tooth surfaces, pH-responsive drug release, and more powerful destruction to biofilm than the antibacterial agent alone.

pH is a critical risk factor to keep the balance of dental biofilm micro-ecological environment (Pitts et al., 2017; Bowen et al., 2018). In neutral pH, various bacteria colonize the tooth surface together and maintain a biofilm environment that is compatible with health. On exposure to an abundant supply of carbohydrates, large amounts of acidic bacterial metabolites will be produced and a low pH environment in biofilm (pH 4.5–5.5) will be formed (Vroom et al., 1999; Takahashi, 2005). This acidic environment will suppress bacteria that have a low acid resistance and allow bacteria with high acid resistance to flourish and produce more acids, thereby causing tooth demineralization and development of dental caries (Hwang et al., 2016; Bowen et al., 2018). Hence, delivering effective antibacterial agents into mature biofilm, which has an acidic environment, to act on the cariogenic bacteria will be a good caries prevention strategy. Currently, many pH-responsive drug delivery nano-materials have been explored in acidic pathological environments, such as cancer microenvironment, bone infections, and *Pseudomonas aeruginosa* infections (Cicuéndez et al., 2018; Naha et al., 2019; Chen et al., 2021). However, relatively limited research has been conducted on their application in the prevention of dental caries (Horev et al., 2015; Zhao et al., 2019; Liang et al., 2020).

Bedaquiline is an inhibitor that targets an acid-resistant protein H^+^-ATPase (Preiss et al., 2015). Results of our previous study show that bedaquiline can potently inhibit the growth of *Streptococcus mutans* (*S. mutans*) suspension and biofilm generation in acidic environment (pH 5) but has no effect in neutral environment (Zhang et al., 2021). Besides, this hydrophobic agent cannot exert bactericidal effect on the bacteria within biofilm. Subsequently, in order to enhance the bactericidal effect of bedaquiline in mature biofilms, and to promote the clinical application of hydrophobic antibacterial agents in caries prevention, an effective carrier such as pH-responsive drug delivery nano micelle needs to be exploited to tackle this challenge.

Therefore, in the present study, a pH-responsive nano micelle [methoxypolyethylene glycol-b-poly-2-(diisopropylamino) ethyl methacrylate (mPEG-b-PDPA)] with and without bedaquiline loading was synthesized using ATRP (atom transfer radical polymerization). Its physical characterization was carried out by Hydrogen-1 nuclear magnetic resonance (^1^H-NMR), gel permeation chromatography (GPC), critical micelles concentrations (CMC), dynamic light scattering (DLS), and transmission electron microscopy (TEM). The cytotoxic effect on the periodontal cells and the antibacterial effect of micelles on *S. mutans* and the bactericidal effect on mature *S. mutans* biofilm were determined.

## Materials and Methods

### Materials

Methoxypolyethylene glycol-OH (mPEG, MW2000); α-bromoisobutyryl bromide (EBiB, 98%, 195.05 g mol^–1^); 2,2-dipyridyl (bPy, 99%, 156.19 g mol^–1^); cuprous bromide (CuBr, 99%, 143.15 g mol^–1^); N,N,N′,N′,N′-pentamethyldiethylenetriamine (PMDETA, 173.3 g mol^–1^); dichloromethane (DCM); triethylamine; tetrahydrofuran (THF); and phosphotungstic acid were purchased from Macklin (Shanghai, China). 2-(Diisopropylamino) ethyl methacrylate (DPA) was purchased from Sigma-Aldrich (Merck KGaA, German). Bedaquiline was purchased from MedChemExpress (United States).

### Synthesis of Methoxypolyethylene Glycol-Br Macroinitiator

The preparation of mPEG-Br macroinitiator followed the method in a previous study with some modifications as described below (Yu et al., 2011). mPEG (2 g, 1 mmol) was dissolved in 10 ml of DCM in a Schlenk flask followed by the addition of triethylamine (416 μl, 3 mmol). α-Bromoisobutyryl bromide (371 μl, 3 mmol) was quickly dropped into the mPEG DCM solution and immediately sealed for 2 h in ice bath. The reaction continued overnight at room temperature. The product was purified by filtration, rotary evaporation, dialyzing (MWCO 1000 Da) against Milli-Q water, and dried by lyophilizing.

### Synthesis of Methoxypolyethylene-b-Poly-2-(Diisopropylamino) Ethyl Methacrylate Diblock Copolymer *via* Atom Transfer Radical Polymerization

The synthesis of mPEG-b-PDPA diblock copolymer essentially followed the method in previous studies (Du and Armes, 2005). Macroinitiator mPEG-Br (430 mg, 0.2 mmol) and 2,2-dipyridyl (62.5 mg, 0.4 mmol) were dissolved in 35 ml of DMF/DI water mixture (v/v 4:1, Schlenk flask), stirring 15 min in an ice bath. DPA (4.74 ml, 20 mmol) was added and immediately degassed by three nitrogen cycles. Anhydrous CuBr (290 mg, 2 mmol) was immediately added and degassed by three nitrogen cycles. The polymerization was carried out at 40°C for 12 h. The product was purified by dialyzing against Milli-Q water (MWCO 3500 Da). Two milligrams of PMDETA was added to the first few dialysis cycles to replace the Cu ions in the product and the Cu ions were removed until no blue product precipitated. Then the product was dialyzed for another 3–5 cycles and dried by lyophilizing.

### Preparation of Methoxypolyethylene Glycol-b-Poly-2-(Diisopropylamino) Ethyl Methacrylate Micelles

Typically, 10 mg of mPEG-b-PDPA diblock copolymer was dissolved in 1 ml of THF. The mPEG-b-PDPA micelles were formed by adding the solution into 4 ml PBS (pH 7.4, 1 mmol L^–1^) dropwise, while stirring it for 12 h to volatilize the THF. The micelles were further purified by dialyzing against Milli-Q water (MWCO 3500 Da) and the precipitate was collected by centrifugation (14,000 rpm, 10 min, repeat 4 times). The storage micelle dispersion solution was prepared by adding 400 μl Milli-Q water (free micelles: 25 mg ml^–1^ mPEG-b-PDPA). The bedaquiline-loading micelles (10 mg ml^–1^ bedaquiline if it was completely encapsulation) were prepared as follows. In brief, 4 mg of bedaquiline was dissolved in 1 ml THF solution containing 10 mg of mPEG-b-PDPA diblock copolymer, followed by adding this solution dropwise into 4 ml PBS. The following protocol was exactly the same as those of mPEG-b-PDPA micelles’ synthesis.

### Structural Characterizations of Polymers and Micelles

The ^1^H-NMR spectra of mPEG-Br and mPEG-b-PDPA dissolving in D_2_O and CD_2_Cl_2_ were recorded with Bruker AVANCE III HD 400 MHz. The molecular weight and polydispersity index (PDI) of mPEG-b-PDPA polymer were acquired from GPC, THF was selected as the eluent. The hydrodynamic size diameter and zeta-potential of free micelles and bedaquiline-loading micelles at different pH values (5–7.4) were measured by dynamic light scattering (DLS, Zetasizer μV, Malvern).

### pH Titration of Methoxypolyethylene Glycol-b-Poly-2-(Diisopropylamino) Ethyl Methacrylate Copolymers

The mPEG-b-PDPA copolymer was first dissolved in 0.1 N (0.1 mol L^–1^) HCl to prepare a stock solution at a concentration of 5 mg ml^–1^. pH titration was carried out by adding 20 μl increments of 0.1 N (0.1 mol L^–1^) NaOH solution under stirring. The increase in pH in the range of 3–8 was monitored as a function of the total added volume of NaOH (V_NaOH_). The pH values were measured using pH meter.

### Measurement of Critical Micelles Concentrations

The CMC of the free micelles was determined by surface tension (platinum Wilhelmy plate). A set of solutions (1 ml) with different copolymer concentrations were dropped into 20 ml PBS under stirring to prepare the micelles solutions. The surface tension of micelles solutions was measured using Force Tensiometer (Sigma 700/701, Biolin Scientific, Sweden) and the CMC were calculated using the software OneAttension.

### Bedaquiline-Loading Ratio and Encapsulation Efficiency

Ten microliters of the bedaquiline-loading micelles and free micelles were dissolved in 1 ml of THF solution. The absorption (309_n__m_) of these two solutions was determined by UV-Vis spectra. Based on the standard curve of UV-Vis absorption of a set of bedaquiline THF solutions with defined concentrations, the bedaquiline-loading weight (m3) was determined. The bedaquiline-loading ratio and encapsulation efficiency were calculated as follows:


Loadingratio=m3/m1×100%

Encapsulationefficiency=m3/m2×100%

where m1 is the weight of the nanoparticles, and m2 is the total weight of bedaquiline fed.

### Transmission Electron Microscopy Imaging of Free Micelles

The storage micelles diluted 10^6^ times were negatively stained using 1% freshly prepared phosphotungstic acid (w/v%, PBS, pH 7), and then dropped on the copper grid and dried. The images of micelles were captured under TEM.

### The Cumulative Release of Bedaquiline in Different pH Buffer

The 0.1 M PBS buffers of pH 5, 6, and 7.4 were prepared. Ten microliters of the bedaquiline-loading micelles and free micelles were separately mixed in 1 ml of PBS buffers with different pH. The release of bedaquiline was determined by UV-Vis spectra (λ = 309_n__m_) until the cumulative release reached close to 100%.

### The Antibacterial Activity of Micelles

Both the bacterial killing and the antibiofilm formation efficacy of micelles and bedaquiline-loaded micelles in pH 5 and 7 condition were assessed. The fourfold dilution of the storage micelle solution was prepared as working solution [if there was no loss (ideal situation): free micelles, 6 mg ml^–1^ mPEG-b-PDPA; bedaquiline-loaded micelles, 2.5 mg ml^–1^ bedaquiline]. In brief, study solutions containing 1, 0.5, or 0.1% working solution were mixed with BHI medium at pH 5 or 7 in a sterile 96-well plate and incubated with *Streptococcus mutans* UA159 (ATCC10449; mid-logarithmic stage, 10^6^ CFU ml^–1^) under anaerobic condition at 37°C for 24 h. For the BHI medium for biofilm formation, 1% (w/v%) of sucrose solution was added at a ratio of 1:100. Then the absorption value at 600_n__m_ was measured to assess bacterial growth.

### Live/Dead Bacteria Staining

*Streptococcus mutans* UA159 was incubated in BHI containing 1% sucrose at 37°C for 24 h in sterile 96-well microplates to form a mature biofilm on cover glass. Then different ratios of micelle solutions were mixed with fresh BHI (pH 7.4) and applied onto the biofilms. The different solutions contained were sterile Milli-Q water, 1, 0.5, and 0.1% working solutions, including free micelles and bedaquiline-loading micelles (corresponding bedaquiline-loading concentrations: 25, 12.5, and 2.5 μg ml^–1^). After 2 h, the cover glasses with biofilm were gently washed with PBS to remove the unattached bacteria and stained with SYTO9 and propidium iodide for 30 min (Invitrogen^TM^, United States). The stained samples were imaged with Olympus Fluoview FV1000.

### Cytotoxicity Assay

The *in vitro* cytotoxicity of micelles was evaluated by CCK-8 assay using periodontal ligament stem cells (PDLSCs, P5). In brief, PDLSCs (5,000 cells/well) were seeded in 96-well microplates and incubated at 37°C for 24 h. Subsequently, the medium was replaced by fresh culture media containing 1, 0.5, and 0.1% working micelles solutions for further cultivation of 24 and 48 h. Then cell counting kit-8 (CCK8, DOJINDO, Japan) solution was added into each well and incubated in the dark at 37°C for 2 h. Finally, the absorption values were measured by a microplate reader at 450_n__m_. Five parallel groups were used for each group.

### Statistical Analysis

Each of the above-described tests was repeated three times to generate data for analysis. All data were statistically analyzed using SPSS Statistics v17 and the figures were drawn using GraphPad. *T*-test and one-way analysis of variance (ANOVA) were performed to assess the statistical significance of the differences in means among groups, with the significance level set at 0.05.

## Results and Discussion

### Synthesis and Characterization of Polymers

**FIGURE 1:**
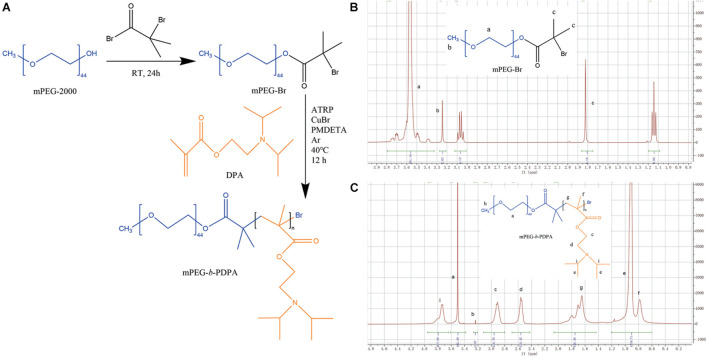
Synthetic routes of methoxypolyethylene glycol-b-poly-2-(Diisopropylamino) ethyl methacrylate (mPEG-b-PDPA) **(A)** and hydrogen-1 nuclear magnetic resonance (^1^H-NMR) spectra of mPEG-Br in D_2_O **(B)** and PEG-b-PDPA in CD_2_Cl_2_ **(C)**.

In this study, well-defined poly (ethylene glycol)-block-poly [2-(diisopropylamino) ethyl methacrylate] (mPEG-b-PDPA) diblock copolymers were obtained *via* atom transfer radical polymerization (ATRP, see Figure 1A). The copolymer had polymer molecular weights Mw: 20,941 and Mn: 16,271 as measured by GPC, and polydispersity indices (PDI) of 1.28 (Table 1). The ^1^H-NMR spectra of PEG-Br and mPEG-b-PDPA are shown in Figures 1B,C. The methyl protons of the α-bromoisobutyryl bromide group of PEG-Br showed the characteristic chemical shift at 3.562 ppm, which indicates the successful synthesis of PEG-Br macroinitiator (Figure 1B). The chemical shift at 1.120 and 3.060 ppm was the unreacted triethylamine. It has been removed by the repeat dialysis. For the spectra of mPEG-b-PDPA (Figure 1C), the newly appeared chemical shifts located at 0.75, 0.92, 1.65, 2.52, 2.90, and 3.73 ppm were due to the resonance of PDPA. The integrals ratio of PEG block and PDPA block was similar to the added molar ratio (0.2:20 mmol), and the estimate was 44:(102–112). These test results verified the successful synthesis of mPEG-b-PDPA copolymer with low polydispersity. Compared with the synthesis of other pH-responsive nanopolymers, the synthesis of mPEG-b-PDPA is simple (Horev et al., 2015; Deirram et al., 2019; Naha et al., 2019; Zhao et al., 2019; Liang et al., 2020).

**TABLE 1:** Molecular weight results of mPEG-b-PDPA polymer based on GPC.

Composition	Mw	Mn	PDI	CMC (mg L^–1^)
mPEG-b-PDPA	20,941	16,271	1.28	3.8

*mPEG-b-PDPA, methoxypolyethylene glycol-b-poly-2-(diisopropylamino)ethyl methacrylate; GPC, gel permeation chromatography; PDI, polydispersity index; CMC, critical micelles concentrations.*

**FIGURE 2:**
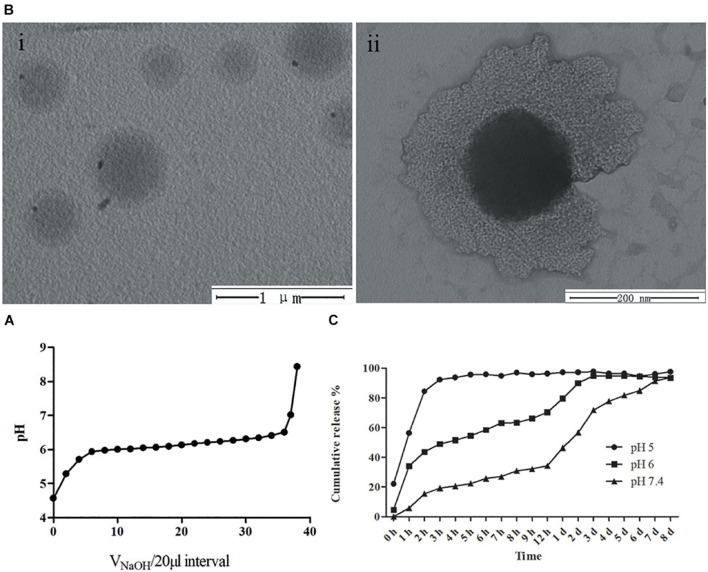
**(A)** pH titration curve of mPEG-b-PDPA polymer; **(B)** transmission electron microscopy (TEM) image of free micelles at pH 7 **(i)**, and typical core-shell structure image of micelle **(ii)**. **(C)** Cumulative drug release curves of bedaquiline-loading micelles at pH 5, 6, and 7.4 PBS buffer.

In addition, pH titration results of mPEG-b-PDPA copolymers (Figure 2A) showed that when NaOH was continuously added dropwise, a sharp turn of pH curve occurred at pH 6 and remained at this level. This phenomenon is probably because of the deprotonation of DPA tertiary ammonium groups (Zhou et al., 2011), and is in agreement with the acid dissociation constant of PDPA segment (p*Ka* = 6.3) (Yu et al., 2011).

The UV-Vis spectra results showed that the bedaquiline-loading weight (m3) was 3.7 mg. It was calculated that 1 ml of storage micelles contained *ca.* 9.25 mg bedaquiline. The bedaquiline-loading ratio and encapsulation efficiency of mPEG-b-PDPA were calculated as 37 and 92.5%, respectively (m1 = 10 mg, m2 = 4 mg). This micelle displays a good hydrophobic agent encapsulation capacity (Horev et al., 2015; Qing et al., 2019).

### Physical Characterization of Micelles

mPEG-b-PDPA micelles were prepared with and without bedaquiline-loading. First, we measured the diameter of micelles in different pH aqueous solution using DLS (Table 2). All micelles showed relatively narrow size distribution (polydispersity < 0.3). At pH 7.4, free micelles had a diameter of around 322.4 ± 2.69, while the diameter of bedaquiline-loaded micelles slightly decreased (260.2 ± 2.02, *p* < 0.05). At pH 6, the diameter of micelles with and without bedaquiline-loading increased significantly (1,133.0 ± 40.43 and 905.8 ± 35.73), and the diameter could not be measured at pH 5.

**TABLE 2:** Particle size and zeta potential of free micelles and bedaquiline-loading micelles based on DLS.

		Free micelles	Bedaquiline-loading micelles
pH 7.4	Size	322.4 ± 2.69	260.2 ± 2.02
	PDI	0.146 ± 0.03	0.145 ± 0.02
	ζ	39.8 ± 0.45	47.1 ± 0.74
pH 6	Size	905.8 ± 35.73	1133.0 ± 40.43
	PDI	0.248 ± 0.03	0.071 ± 0.01
	ζ	4.97 ± 1.03	–0.09 ± 0.15
pH 5	Size	–	–
	PDI	–	–
	ζ	47.8 ± 2.84	–0.36 ± 0.57

*mPEG-b-PDPA, methoxypolyethylene glycol-b-poly-2-(diisopropylamino)ethyl methacrylate; GPC, gel permeation chromatography; PDI, polydispersity index; CMC, critical micelles concentrations; DLS, dynamic light scattering.*

In this study, the changes of nanometer size in different pH solutions are consistent with the pH-responsive property of PDPA and the pH titration results. When the environmental pH drops below the p*K*_*a*_ (pH < 6), the protonation of PDPA segment shifts the hydrophobic in the core to hydrophilic, the drug is released, and the spherical nano structure of micelle swells and even disassembles (Deirram et al., 2019). This property of regulating drug release through protonation and deprotonation is shared by pH-responsive materials (Belkova et al., 2005; Horev et al., 2015; Bazban-Shotorbani et al., 2017), which makes them regulate drug release in a more precise environment. The PDPA segment selected in this study can start to release the drug in an environment below pH 6, which enables the drug to enter the local cariogenic environment at a good opportune moment and to exert its bactericidal effect. This property is important for clinical application because the initiation of dental decay occurs in the acidic environment between pH 4.5–5.5 caused by acid-resistant pathogens (Bowen et al., 2018).

The spherical core-shell morphology of the free micelles is shown in the TEM images, especially in Figure 2Bii. However, the diameter of nano micelles in the TEM images is larger than the result of DLS, which may be related to the treatment of phosphotungstic acid. During the sample preparation process, the prepared neutral phosphotungstic acid solution may have been slightly acidified, resulting in swelling of the nanomicelles.

The size of the micelles in this study is 300_n__m_, which is larger than that of the micelles synthesized by other researches (Horev et al., 2015; Zhao et al., 2019). The micelle itself usually has no antibacterial effect (Natan and Banin, 2017; Qing et al., 2019; Zhao et al., 2019), but its size may affect its ability to penetrate into biofilm, its drug loading, and its encapsulation ability. So far, no articles report on the influence of micelle size on its antibacterial ability. This is different from the metal nano materials (Natan and Banin, 2017; Paula and Koo, 2017), for example, the size of silver nanoparticles significantly affects its antibacterial capability, and nanoparticles with smaller sizes generally show greater antibacterial effects (Choi et al., 2008; Sotiriou and Pratsinis, 2010). So, it is necessary to explore the influence of micelle size on its properties in future studies.

In addition, zeta potentials results showed that the micelles in aqueous solution were positively charged under neutral and acidic environments (Table 2). At pH 7.4, the zeta potential of both the free micelle group (ζ = 39.8 ± 0.45 mV) and the bedaquiline-loaded micelle group (ζ = 47.1 ± 0.74 mV) exceeded 30 mV. This indicates that the synthesized micelles were colloidally stable at pH 7.4 (Pate and Safier, 2016), which is a vital property of micelles. The free micelles group at pH 5 was also stable, with a zeta potential at 47.8 ± 2.84 mV. However, the zeta potentials (absolute value) of the free micelles at pH 6 and the bedaquiline-loading micelles at pH 5–6 were lower than 5 mV, indicating these micelles were unstable. These findings are consistent with the results of size detection and pH-response characterization. The incomplete protonation of PDPA segment in acidic milieus (pH 6) triggered the swelling of micelles and turned the micelles into an unstable state. With the complete protonation of PDPA segment at pH 5 the micelles dissembled, i.e., not existing as a core-shell structure, but became stable. In addition, the loaded hydrophobic agent had a negative effect on the stability of micelles in a pH 5 environment.

It should be noted that the micelles in neutral environment are positively charged, which may contribute to the capability of micelles to coat tooth surface and promote bacterial adhesion by electrostatic adsorption. Previous studies found many factors, including energy, charge, composition, stiffness, and hydrophobicity of tooth surface (Belas, 2014; Song et al., 2015, 2017; Teschler et al., 2015), as well as size, charge, and ionic strength of micelles (Paula and Koo, 2017; Narayanan et al., 2020; Li and Liu, 2021), can affect the binding between micelles and tooth surfaces. These binding-promoting properties of micelles should be further studied to improve their clinical application potential.

### Bedaquiline Release of Micelles

**SCHEME 1:**
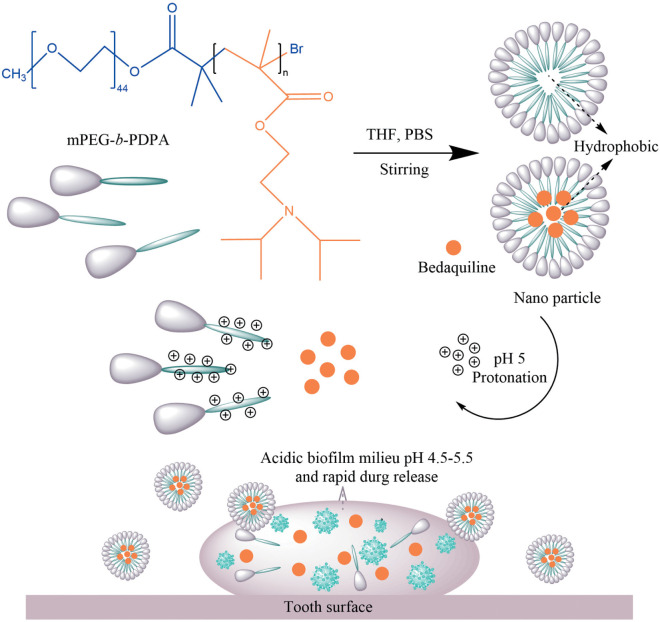
Scheme Chemical formulas of diblock copolymer (mPEG-b-PDPA); Schematic representation for the formation and pH-responsive property of micelles; and the proposed mode of pH-responsive of micelles for biofilm penetration property.

Bedaquiline release from the micelles is displayed in Figure 2C. At pH 7, the rate of release of bedaquiline was very gentle, not exceeding 35% in the first 12 h, and the cumulative release reached over 90% only after 8 days. At pH 6, the amount released within 12 h reached 70.3%, and this reached 94.8% on the third day. At pH 5, the amount released reached 92.2% in 3 h. These findings show the bedaquiline-release of mPEG-b-PDPA mainly relay on the pH-sensitive properties of PDPA block (Yu et al., 2011, 2013). Under neutral pH conditions, the PDPA block is hydrophobic, and the hydrophilic PEG surrounds the PDPA to form a core-shell structure. Hydrophobic agents such as bedaquiline can be encapsulated by the micelle. When the micelle is put in acidic environments (pH < 6), protonation of the PDPA block converts it into hydrophilic. Then, the micelle will gradually swell and the core-shell structure will dissemble, releasing the bedaquiline in the core (see the Scheme 1). This is the most common mechanism of pH-responsive nanocarrier materials. This kind of pH-responsive property allows the encapsulated drug to be released rapidly and massively in a local acidic environment (Yu et al., 2011; Liu et al., 2016; Bazban-Shotorbani et al., 2017; Deirram et al., 2019), which greatly improves the local working concentration and efficiency.

### Antibacterial and Anti-biofilm Formation Effect of the Study Micelles

**FIGURE 3:**
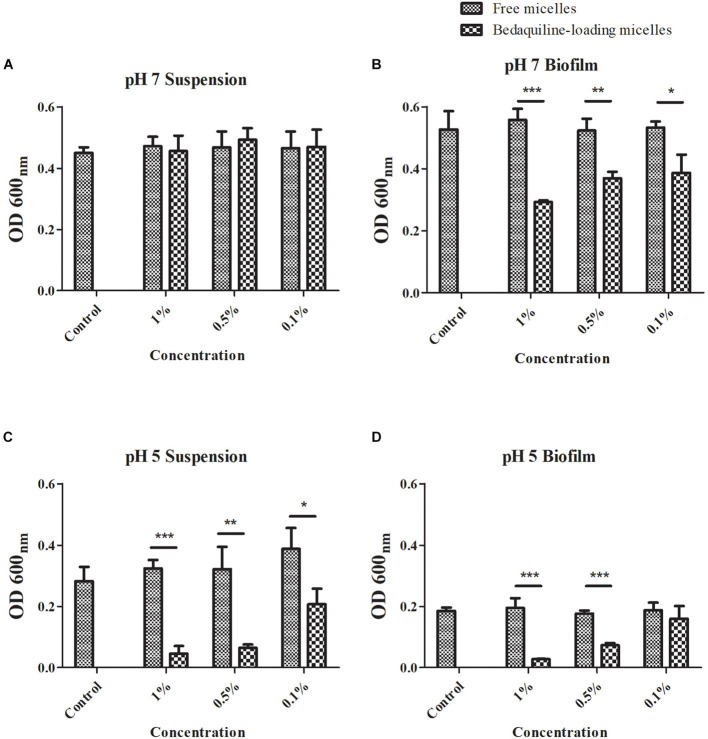
Antibacterial effect of free micelles and bedaquiline-loading micelles at pH 7 and 5 BHI broth. At pH 7, antibacterial effect of free micelles and bedaquiline-loading micelles at 0.1, 0.5, and 1% ratio (v/v%) against **(A)** *Streptococcus mutans* suspension and **(B)** biofilm formation. At pH 5, antibacterial effect of free micelles and bedaquiline-loading micelles against **(C)** *S. mutans* suspension and **(D)** biofilm formation. *P < 0.05, **P < 0.005, and ***P < 0.001.

The antibacterial effect of the study micelles with and without bedaquiline loading on *S. mutans* in pH 5 and 7 are summarized in Figure 3. Compared with the control group, at pH 7, the growth of *S. mutans* suspension in all the free micelles groups and the bedaquiline-loaded micelles groups were not inhibited (Figure 3A). In the *S. mutans* biofilm model, free micelles had no inhibitory effect on biofilm formation at pH 7 (Figure 3B), while the inhibitory effect of the bedaquiline-loaded micelles increased with the concentration of bedaquiline-loaded micelles (*p* < 0.05). Among them, the 1% bedaquiline-loaded micelles group (contain *ca*. 25 μg ml^–1^ bedaquiline) reduced the growth rate of the bacteria to half that of the control (*p* < 0.001). Our pervious study had found that at pH 7, bedaquiline cannot inhibit the biofilm formation of *S. mutans* (Zhang et al., 2021). In order to figure out the reasons for these inconsistent results, we had conducted additional study to measure the pH change of medium during bacterial growth. It was found that without any intervention, when *S. mutans* planktonic bacteria were cultured for 24 h at pH 7, the pH of the medium dropped to about 5.8, while in the model where extra 1% sucrose was added to construct the *S. mutans* biofilm, the pH of the medium dropped to about 4.9 (data not shown). Therefore, we speculate that during biofilm development, when the pH of biofilm internal environment dropped to about 5, the nano micelles that had penetrated into the biofilm released the encapsulated bedaquiline and exerted a bactericidal effect. However, the pH of the suspended bacterial medium did not reach the level at which the encapsulated bedaquiline would be released, so no inhibitory effect was detected in the suspension groups. The inhibitory effect of bedaquiline-loaded micelles on *S. mutans* biofilm formation at neutral environment also indicates that the study nano micelles can penetrate into the biofilm and release antibacterial agent in an acidic environment.

When *S. mutans* was incubated in an acidic environment, the acidic environment itself had an inhibitory effect on the bacterial growth (see the control group in Figures 3A–D). At pH 5, bedaquiline-loaded micelles further significantly inhibited the growth of planktonic bacteria and also the formation of biofilms (Figures 3C,D). In the model of *S. mutans* suspension at pH 5 (Figure 3C), the free micelles groups did not show a significant antibacterial effect. While in the 0.1% bedaquiline-loaded micelles group [contain *ca.* 2.5 μg ml^–1^ bedaquiline (IC_50_)] the growth of *S. mutans* in suspension was significantly reduced to half (*p* = 0.015). The growth of *S. mutans* in the 0.5% and the 1% bedaquiline-loaded micelles groups was even lower (*p* = 0.003 and *p* < 0.001). In the *S. mutans* biofilm model at pH 5 (Figure 3D), compared with the control, the free micelles groups and the 0.1% bedaquiline-loaded micelles group had no antibacterial effect (*p* > 0.05) while the 0.5% and the 1% bedaquiline-loaded micelles groups significantly inhibited the development of biofilm (*p* < 0.001).

### Bacterial-Killing Effects of Bedaquiline-Loading Micelles Against Mature *Streptococcus mutans* Biofilm

**FIGURE 4:**
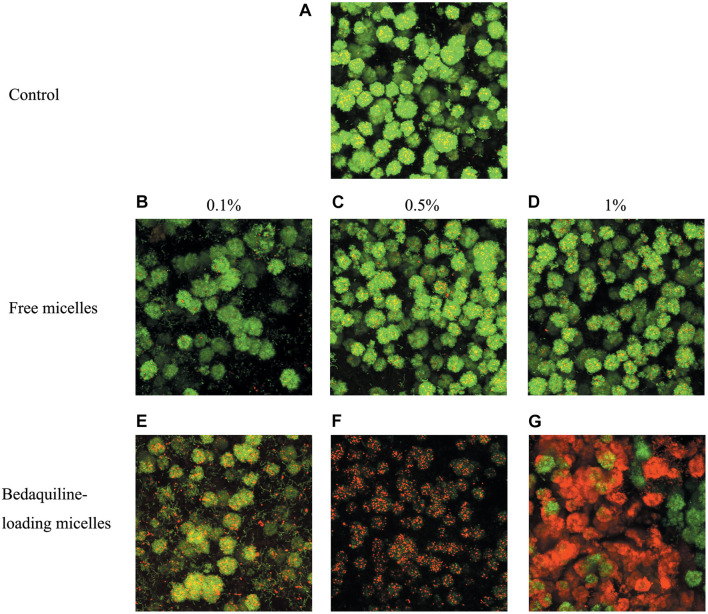
Live/dead bacterial staining of *S. mutans* biofilm. Mature biofilm was shocked for 2 h by BHI broth (positive control **A**), free micelles at 0.1% **(B)**, 0.5% **(C)**, 1% **(D)** ratio (v/v%), and bedaquiline-loading micelles at 0.1% **(E)**, 0.5% **(F)**, 1% **(G)** (v/v%). Green, live bacteria; red, dead bacteria; yellow, merged image of live and dead bacteria.

The live/dead bacteria staining imaging of mature biofilms treated with micelles are shown in Figure 4. The images in the control and the free micelle groups, presence of dead bacteria (red staining) was not obvious. In contrast, the bedaquiline-loaded micelles had bactericidal effects which increased with the micelle concentration. In the 0.5% and the 1% groups, most of the bacteria in the biofilm were dead.

The stable and cohesive biofilm formed by *S. mutans* (Cross et al., 2006; Koo et al., 2013) can block the penetration of antibacterial agents, including bedaquiline (Zhang et al., 2021). This is a key self-protection property of biofilms. Dental biofilm mainly relies on exopolysaccharides to strengthen the interconnection between bacterial cells (Koo et al., 2013; Lin et al., 2021). Bedaquiline has no influence on the carbohydrate metabolism of *S. mutans*, while it can bind to H^+^-ATPase and destroy the cell membrane, exerting a bactericidal effect (Zhang et al., 2021). The bactericide effect on mature biofilm found in the present study shows that the study nanomicelle drug delivery system facilitates the penetration of bedaquiline into bacterial biofilm, making the contact between bedaquiline and bacteria available, and then killing the bacteria inside the biofilm. Theoretically, any hydrophobic agents can be loaded by this nanocarrier system (mPEG-b-PDPA).

### Cytotoxicity Assay

**FIGURE 5:**
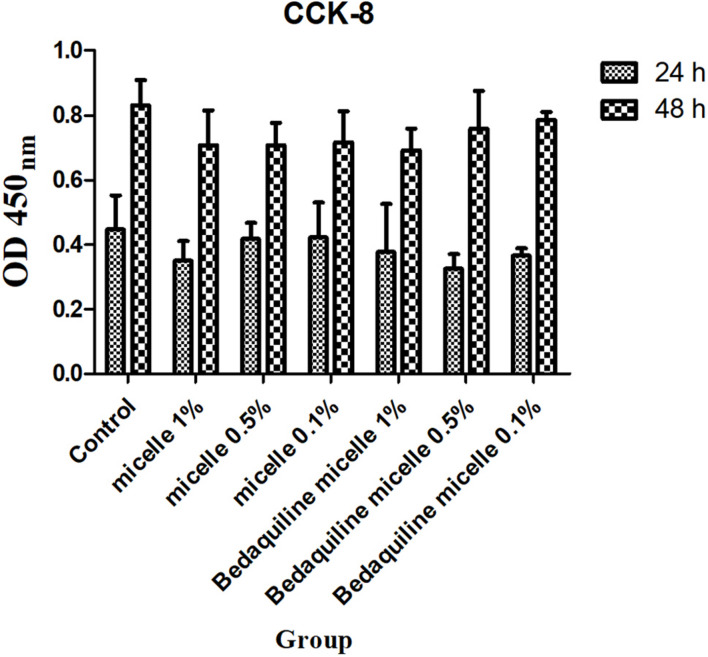
Cytotoxicity assay of free micelles and bedaquiline-loading micelles at different concentrations.

To study the safety of this pH-responsive nanocarrier system, 48-h cytotoxicity assay of micelles on PDLSCs cells was evaluated. As shown in Figure 5, both micellar nanocarrier groups (unloaded and bedaquiline-loaded) did not exhibit significant cytotoxicity against PDLSCs cells at 24 and 48 h. Therefore, this pH-responsive nanomicelle is biocompatible and can be used safely for hydrophobic antibacterial drug delivery (Zhao et al., 2019).

### Limitation of the pH-Responsive Nanocarrier Micelles

The study pH-responsive nanocarrier micelle, mPEG-b-PDPA, is simple to synthesize and can encapsulate hydrophobic antibacterial agents. Its good performance on antibacterial and bactericidal effects against *S. mutans* suspension and biofilm shows its clinical application potential. However, there are some limitations. First, the solvent used to prepare the micelles needs to be applicable for the hydrophobic antibacterial agents and be easy to remove. Second, due to the agent-loading to micelles weight ratio has not been optimized, this limits its application for hydrophobic antibacterial agents with high MIC values (over than 100 μg/ml). Third, this micelle-loading agent is a one-time release system, it is difficult to re-encapsulate agents and form micelles when the pH returns to neutral. Hence, a reversible micellar system capable of loading antibacterial agent needs further exploration.

## Conclusion

To sum up, in this study a two-block micellar nanocarrier, mPEG-b-PDPA, was synthesized using a simple ATRP method. The micelle surface is positively charge and can be loaded with hydrophobic drugs. The bedaquiline-loaded nano-micelles inhibit the growth of *S. mutans* in neutral and acidic environments, and have a good bactericidal effect on mature biofilm. Furthermore, it does not have any cytotoxic effect on periodontal cells. The study findings show that it has a good potential for clinical application in dental caries prevention.

## Data Availability Statement

The original contributions presented in the study are included in the article/supplementary material, further inquiries can be directed to the corresponding author.

## Author Contributions

MZ, ZY, and EL conceived this study and analyzed the data. MZ conducted the experiments and wrote the manuscript. All authors contributed to the article and approved the submitted version.

## Conflict of Interest

The authors declare that the research was conducted in the absence of any commercial or financial relationships that could be construed as a potential conflict of interest.

## Publisher’s Note

All claims expressed in this article are solely those of the authors and do not necessarily represent those of their affiliated organizations, or those of the publisher, the editors and the reviewers. Any product that may be evaluated in this article, or claim that may be made by its manufacturer, is not guaranteed or endorsed by the publisher.
